# Digitized diaspora governance during the COVID‐19 pandemic: China's diaspora mobilization and Chinese migrant responses in Italy

**DOI:** 10.1111/glob.12389

**Published:** 2022-07-25

**Authors:** Antonella Ceccagno, Mette Thunø

**Affiliations:** ^1^ Department of Modern Languages, Literature and Cultures University of Bologna Bologna Italy; ^2^ Department of Global Studies Aarhus University Aarhus Denmark

**Keywords:** Chinese migrants, COVID‐19, diaspora engagement, digital diaspora governance, Italy, WeChat

## Abstract

We explore how the Chinese diaspora state during the COVID‐19 pandemic in 2020 managed to transform a severe health crisis into a geo‐political opportunity for transnational nation‐building through diaspora governance based on extensive use of social media technologies. By adopting a multi‐scalar perspective, we analyse the intertwined nature of top‐down and bottom‐up processes of the Chinese Party‐state's diaspora mobilization. Based on discourse and ethnographic analysis, we argue that China's diaspora governance exposed a new and strong capacity for extra‐territorial governance. We explore how discursive hegemony, social control and diaspora mobilization were achieved by widely employing the Chinese social media application, WeChat. We also contend that this was facilitated by the Italian government's and media's pro‐China attitudes to emphasize the importance of considering transnational embeddedness when studying the implementation and impact of interactive online technology for diaspora governance in an illiberal political context.

## INTRODUCTION

The Chinese government's domestic containment of COVID‐19 has received media scrutiny and scholarly attention, but there has been much less focus on a crucial development favoured by the pandemic: China's enhanced transnational governance of the Chinese diasporas. The COVID‐19 pandemic that developed into a global health and political crises for China in 2020 offers an exceptional opportunity to analyse how the Chinese Party‐state governed Chinese diasporas under intense international and domestic pressure, and how Chinese migrants and their descendants responded.

In this article, we explore how Beijing managed the COVID‐19 crisis regarding ‘its’ population abroad, and how this has strengthened the relationship between the Chinese Party‐state and the Chinese diasporas. We adopt a multi‐scalar perspective by addressing (i) official Chinese political discourses presented at the national scale targeting the Chinese diasporas; (ii) the mobilization of institutions and resources at the local scale in China; and (iii) the agency of transnational Chinese in Italy, in terms of consenting to actively using the Chinese state‐controlled social media platform WeChat. By focusing on national, local and transnational scales, we reveal the intertwined nature of top‐down and bottom‐up processes of digital diaspora governance and engagement.

To analyse the reactions of Chinese populations overseas to China's diaspora governance, we selected Italy with 318,003 Chinese nationals (Ministero del Lavoro e delle Politiche Sociali, 2020). This makes Italy the country with the highest official number of residing Chinese nationals in the European Union, and Italy also became the first major European country to sign a Memorandum of Understanding related to China's Belt and Road Initiative in 2019. During 2020, the Italian government thus pursued a policy of closer ties with China in contrast to other major European countries.

As this paper shows, Italy's political position vis‐à‐vis China in 2020 had an impact on the Italian government and media narratives of the pandemic, and consequently on Chinese nationals and ethnic Chinese residing in Italy. This makes Italy a crucial setting in Europe for analysing how Chinese nationals living in a country with a government friendly to China responded to China's diaspora governance in a time of crisis.

We argue that Beijing's transnational management of the COVID‐19 crisis has exposed a significant capacity for extra‐territorial mobilization, and the Chinese Party‐state's ability to turn a severe health and political crisis into a transnational political tool. This was made possible not only through the mobilization of Beijing's already well‐established bureaucratic diaspora infrastructure, but particularly by using the all‐pervasive social media application, WeChat. This remains an understudied topic, yet digital technology became a crucial tool that during the pandemic enabled the Chinese Party‐state to achieve social monitoring and effectively mobilized Chinese diasporas in many parts of the world. In this paper, we focus on interactive digital technology as a crucial factor for understanding China's recent diaspora governance in relation to Chinese in Italy.

Below, we first conduct discourse analyses of official material that presents the Chinese Party‐state as compassionate and effectively lifesaving, and as discursively defining Chinese overseas as an emblem of transnational unity between the Chinese nation and the Chinese Communist Party (CCP). We contend that these hegemonic discourses came to inform the ideological attitudes, emotions and civil actions of many Chinese in Italy.

By applying ethnographic methods focusing on Chinese nationals and their descendants living in Italy, we offer a preliminary investigation exploring the extent to which China's employment of social media practices to impose social monitoring and state‐based hegemonic discourses were accepted. We argue that by widely consenting to digital monitoring and mobilization, Chinese migrants actively became involved in shaping and reshaping China's new approach to digital diaspora governance.

## CONCEPTUALIZING DIASPORA GOVERNANCE OF AN AUTHORITARIAN SUPERPOWER

Scholarly literature on diaspora governance, policies and strategies has proliferated in tandem with the growing number of states that are actively going beyond their territorial limits to engage transnational relations. Gamlen argues that emigration states ‘govern’ diasporas as a ‘normal form of political organization’ (Gamlen, 2008, p. 842), which implicitly refers to democratic states extending political, civil and social rights to diaspora populations for capacity building (Gamlen et al., 2019); less has been theorized about authoritarian states extending their governing power beyond territorial borders. It has mainly been argued that out of legitimation and security concerns such states restrain and repress overseas dissidents (Dalmasso et al., 2017; Glasius, 2018; Tsourapas, 2018, 2020, 2021).

However, successful illiberal regimes do not solely rely on abuse of power as this usually comes at a high cost, particularly when applied outside of state sovereignty. They also rely on gaining legitimacy by providing a normative foundation for political authority (Svolik, 2012) and by increasing discursive and social inclusion facilitated by digital technologies (Gueorguiev, 2021).

Although the employment of digital technologies by diaspora states to engage transnational populations is quickly developing with the technical advances of internet‐mediated interactions, scholarship on the use and implications of digitized diaspora governance is still in the making (Kang, 2017). Many diaspora states have made a one‐way flow of state digital information available through official websites with policies and information, but more recently diaspora states engage in direct interpersonal ties with transnationals through interactive online application platforms. To understand and explain the interplay between technology and authoritarian diaspora governance, this paper seeks to analyse how discursive and social inclusion, engagement and mobilization, data gathering and monitoring as well as surveillance practices are facilitated and enhanced by digital technologies. From an analytical perspective, the pandemic offers a unique opportunity to explore how the Chinese diaspora state under severe stress applied new methods of diaspora governance on interactive platforms to include, manage, control and mobilize diaspora populations. In this case, WeChat was applied beyond making Chinese language diasporic media available through subscription services. It was also used by local Chinese diaspora agencies in collaboration with local ethnic associations abroad to set up a wide web of WeChat groups facilitating immediate community and personalized communication.

We are cautious to avoid a state‐centric approach that conceptualizes diaspora governance as the state being a macro‐agent launching top‐down initiatives to control, discipline and co‐opt migrants. Diasporas may be managed by emigration states, but they also mobilize and engage in collective action based on their own agency in local and global contextual embeddedness (Koinova, 2018; Koinova & Tsourapas, 2018). Beijing's diaspora policies, governance and practices are as such contingent on a multiplicity of horizontal and vertical social and institutional actors of Chinese transnationals (Liu & van Dongen, 2016). By empirically focusing on China being a powerful diaspora state, we seek to contribute to the conceptualization of authoritarian diaspora states through analysis of the complexities of top‐down as well as local diaspora management intertwined with bottom‐up mobilization of locally embedded Chinese transnationals during the COVID‐19 pandemic in 2020, using Italy as a case study.

## QUALITATIVE DATA AND METHODS APPLIED

In the following, we analyse how the CCP (Chinese Communist Party) constructed itself as worthy of transnational support and loyalty through the production and control of dominant nationalistic and cultural discourses at the outbreak of the pandemic in 2020. We use discourse analysis to examine key Chinese official political documents, Chinese media reports on local Chinese diaspora agencies’ strategies and management of the pandemic situation, as well as Chinese language media targeting Chinese in Italy and official statements by ethnic Chinese associations. In addition, we consulted Italian language media in 2020. Data and material were collected from January 2020 to December 2020.

The social composition of the Chinese population in Italy is diverse in terms of education, employment, class and age. In the 1990s, first‐generation immigrants from Zhejiang and Fujian provinces found jobs in Chinese‐run workshops in the Italian fashion industry. Some became manufacturers, importers from China and wholesalers (Ceccagno, 2017). Children of Chinese migrants are often involved in diversifying family businesses, even though many seek jobs outside of Chinese circles. A minority of the younger generation have university degrees, and skilled Chinese immigration to Italy is limited.

Between June 2020 and February 2021, we conducted 13 in‐depth, semi‐structured Skype interviews.^1^ While the pandemic imposed some limitations in doing ethnographic research, those who accepted to be interviewed were generous with their time and most interviews lasted between an hour and a half and two and a half hours. One additional interview, lasting two and a half hours, was conducted in June 2022 with one critical interviewee to cross‐check crucial information. Interviewees included ethnic Chinese born in Italy or those having lived in Italy since early childhood, and Chinese studying and working in Italy for some years. Most interviewees were in their 20s and 30s, and men and women were equally represented. Older Chinese emigrants who left China in the 1980s and early 1990s, however, were not represented.

In terms of the interviewees’ professions, there was a computer programmer, two shopkeepers, a lawyer, a restaurant owner, a visual artist, a university professor, a stylist, an engineer and two MA students. Two interviewees had ethnic Italian backgrounds: one working as a school administrator and the other as a civil servant.

Four interviewees held official positions with contacts to large numbers of Chinese: they included a member of a city government council, a co‐founder of an association and two managers of Chinese community websites, one of whom managed some WeChat groups during the pandemic, and the other managed a YouTube programme interviewing among others local Chinese, *Quattro chiacchiere con* (A chat with …). Long conversations with these interviewees make up for the limited number of interviews we were able to make during the pandemic as their central positions enabled them to convey the perceptions and the positioning of many others, including those less represented in our sample. Additional ethnographic research could provide further insights into the topic.

## CHINA MOBILIZING DIASPORAS DURING THE COVID‐19 PANDEMIC

On 25 January 2020, the All‐China Federation of Returned Overseas Chinese (AFROC) under the auspices of the CCP encouraged Chinese diaspora populations to donate money and personal protective equipment (PPE), and to dispatch these to Wuhan, where there was a shortage of medical supplies owing to the outbreak of COVID‐19. Chinese abroad responded swiftly, and large amounts of PPE donations were dispatched by mid‐February to March 2020 (Jia, 2020). According to incomplete official statistics, donations received by 11 March reached RMB 2 billion (Xu, 2020). China National Customs’ clearance figures between 24 January and 29 February 2020 totalled 2.46 billion PPE items worth RMB 8.2 billion, and the Overseas Chinese Charity Foundation of China (Zhongguo huaqiao gongyi jijinhui) announced that by 1 June 2020 it had received RMB 275 million in donations (Prasso, 2020; Ruiz, 2020; Zhongguo Qiaolian Gongyi Jijinhui, 2020).

Although the exact amount of financial and in‐kind donations is not publicly available, there is enough evidence to determine that AFROC's call for Chinese overseas assistance received worldwide positive response from Chinese abroad. Decades of liaison work by state and Party agencies such as AFROC and the Overseas Chinese Affairs Office (OCAO) of the State Council paid off in this situation.

By mid‐March 2020, China had gained better control of COVID‐19, which had by then started to spread to Europe and North America, but as China had no institutional capacity to receive millions of returning emigrants, this led Chinese embassies to discourage Chinese nationals from re‐entering China (Zhu Yidali shiguan, Chinese Embassy in Italy, 2020). More than anything else, the drastically reduced number of flights permitted to land in China led most Chinese nationals to remain in countries that were becoming new focuses of COVID‐19 (Bradsher, 2020).

Instead, the Chinese state immediately began sending PPE supplies to Chinese residing abroad, first to Italy, and later to nearly all countries, as a gesture of compassion. In March, Beijing also started sending medical teams and supplies to many other countries in an attempt to reshape the historical narrative of COVID‐19 in China's favour, as domestic and international criticism escalated.

China's discursive rehabilitation efforts included highlighting the CCP and China as selfless, competent and responsible (Rolland, 2020), and framed its heavy‐handed domestic lockdowns to curb COVID‐19 as in the best interests of the global community (Fallon, 2020). The CCP launched an extensive propaganda campaign to regain legitimacy by emphasizing its leadership in terms of the ‘heroic sacrifices’ of the Chinese people, ‘helping a world in need’ and China becoming ‘a guiding light’ for fighting the pandemic in contrast to liberal democracies (Rolland, 2020).

Beijing's attempts to regain discursive power also among Chinese diaspora populations were strengthened by rising racial discrimination against Chinese and Asian‐looking people in several countries. In an article published in March 2020 in the CCP's political journal, *Qiushi*, Vice Minister of the United Front Work Department (UFWD)^2^ Xu Yousheng commended the deeds of Chinese overseas in terms of rectifying local politicians and journalists, and using petitions and public protests against racial discrimination, exclusion and stigmatization. He also underscored the importance of Chinese abroad being local ‘model students’ by noting the example of the Chinese in Prato, Italy (Xu, 2020).

Calls for Chinese abroad to be law‐abiding and disciplined citizens who extend their assistance locally have been a consistent part of official PRC (People's Republic of China) diaspora policies and discourse. Relying on Chinese diasporas as China's soft power tools is also a well‐known feature of Beijing's more recent diaspora governance (Thunø, 2018), but encouraging Chinese overseas to take to the streets in political, anti‐racist demonstrations is a notable novelty.

Hitherto, appeals to Chinese abroad to serve as public agents by actively protesting in the streets to assert their civil rights in democratic societies had not been officially encouraged in order to avoid jeopardizing China's diplomatic interests and bilateral relationships (Tran & Chuang, 2020). This time, the CCP's willingness to encourage Chinese diasporas to be active agents for its foreign policy goal of rectifying other states’ wrongs against the PRC, including racially motivated attacks, suggests not only the severity of the political repercussions of COVID‐19 as perceived by the Party‐state but also how the Chinese diaspora state succeeded in mobilizing and asserting a strengthened political relationship with the Chinese populations abroad during the crisis situation of the pandemic.

## ‘SENDING CHARCOAL IN SNOWY WEATHER’: REINFORCING PATRIOTISM AND TRANSNATIONAL UNITY

In key political speeches related to the pandemic during 2020, President Xi Jinping stressed the CCP's moral strategy of ‘people coming first and life coming first’ and linked the successful fight against COVID‐19 to the CCP, and thus presenting the Party as not only capable, but also as a benevolent, moral and compassionate ruler (Xi, 2020a, 2020b). Similarly, the UFWD framed China's diaspora management during the pandemic as ‘sending charcoal in snowy weather’ and discursively constructed China as both a lifesaving and caring diaspora state (Xu, 2020).

In official speeches and articles throughout 2020, the ‘patriotism’ and ‘unity’ of the Chinese diasporas with the motherland‐cum‐state became increasingly applied (Renmin Ribao, 2020; Xu, 2020; Zhang & Ran, 2020). Chinese overseas support and proactiveness in the fight against COVID‐19 in China, and later, against the mounting international criticism of China were associated with concepts such as ‘responsibility’, ‘dedication’ and ‘patriotism’ and became a prominent discursive model in official articles (Jia, 2020; Li & Jia, 2020; Renmin Ribao, 2020; Xu, 2020; Zhang & Ran, 2020). Official discourse continuously highlighted the ‘self‐discipline’ and ‘acts of friendship’ of Chinese overseas, such as voluntarily self‐isolation, wearing masks, organizing food delivery and setting up hotlines and emergency centres, so that international communities might learn from Chinese precedent (Xu, 2020).

Official Chinese discourses on Chinese diasporas reflect the CCP's efforts to turn the COVID‐19 health crisis into an opportunity to further make claims on the Chinese diasporas. The circumstances of this severe health crisis thus gave the CCP a discursive platform that was empowered by digital media's amplifications that drew the Chinese diasporas even closer into the orbit of the CCP as we will show below.

## ORGANIZING CARE AND MUTUAL AID GROUPS THROUGH WeChat IN ZHEJIANG AND FUJIAN PROVINCES

At the beginning of 2020, the CCP discursively appealed to the Chinese diasporas’ loyalty and solidarity to seek donations and PPE from overseas, but as the pandemic developed, they also strived to reconcile the situation that Chinese abroad were deterred from returning to China. Thus, we observe how the UFWDs not only intensified the narrative of the CCP as the only reliable political institution and system capable of ensuring health and survival, but in particular they actively mobilized extensive resources to guide and protect the Chinese diaspora abroad.

Reports from UFWDs in Zhejiang and Fujian provinces, from where the majority of Chinese in Europe hail, reflect the tremendous capacity of China's transnational governance infrastructure as digitized diaspora governance was rapidly implemented (Fujian shengwei, 2020; Jia, 2020; Zhejiangsheng Qiaolian, 2020). The prompt mobilization of Chinese businesses, associations and individual Chinese immigrants demonstrated the mature stage of China's diaspora bureaucracy, following its persistent efforts to rebuild and take control of transnational space since the turn of the century (Liu & van Dongen, 2016; Thunø, 2001).

On 4 March 2020, as soon as the Zhejiang division of AFROC published a message to the more than 2 million Zhejiang emigrants and their offspring, most of whom live in Europe, advising them not to return to China, Party Secretary of Zhejiang Province, Che Jun, called a meeting to address deploying transnational epidemic‐prevention and control measures. Within a week, a first batch of 26.4 tons of donated PPE was flown to Italy to local Chinese, local governments and medical institutions, and a week later an expert, 12‐member COVID‐19 medical team arrived in Milan, Italy to provide medical and health assistance to Italian hospitals and resident Chinese. Simultaneously, ‘Zhejiang Internet Hospital Health Care Consultation Platform for Overseas Chinese’ and 700 WeChat groups were launched, which offered migrants from Zhejiang around‐the‐clock online consultations with physicians (Zhejiangsheng Qiaolian, 2020).

Zhejiang AFROC reported that they maintained close contact with the various leaders of Chinese overseas associations, particularly in Italy, Spain and France, to keep them informed and to make sure that the ethnic Chinese associations overseas, such as the chambers of commerce, including the approximately 100,000 Chinese students in their mutual assistance networks, distributed information and forwarded news about established hotlines and PPE. To facilitate liaison with as many overseas Chinese as possible, WeChat groups were established via ethnic Chinese associations abroad in a general e‐matrix structure (Zhejiangsheng Qiaolian, 2020).

Similarly, UFWD and AFROC in Fujian province reported that they reached out to 16 million Chinese migrants, especially in Italy, Japan and South Korea, through 282 ethnic Chinese overseas associations. Measures were taken to enable Chinese migrants to make online sacrifices to their ancestors during the Qingming festival, which usually requires returning to China to sweep the ancestral graves. Medical aid teams were sent to Florence, Italy, 205 around‐the‐clock online health hotlines were established to offer online COVID‐19‐related guidance in Chinese and 26 WeChat groups were set up to offer medical consultations with doctors from Zhejiang who specialized in Western and Chinese medicine (Fujian shengwei, 2020).

The urgent, provincial‐level efforts by Zhejiang and Fujian authorities to extend epidemic protection measures to Chinese abroad reflect the CCP's vast bureaucratic capacity for both analogue and digital organization. Local agencies responsible for Chinese overseas affairs, embassies and consulates were able, at very short notice, to apply social media platforms to connect to millions of Chinese migrants worldwide, through various types of existing elite networks of Chinese migrant and student associations. At the provincial divisions of the UFWD transnational, digitized governance was exercised through the application of WeChat. Applying WeChat as a communication platform facilitated both direct liaison and potential monitoring, down to the level of individual Chinese migrants, in an overseas grid system resembling a domestic digital grid management system (Mittelstaedt, 2022; Pei, 2021).

As the virus was contained in China, the success of digitized diaspora governance in 2020 seemed to have accelerated a pilot project in Qingtian, Zhejiang province, from where many Chinese living in Italy (and Spain) originate. A comprehensive and locally integrated digital platform with information gathering of individual Chinese nationals and transnational communities abroad was integrated with already existing online services within the realm of online courts and police stations (Shu, 2021; W. Xu, 2020; Y. Xu, 2018). With access to personal data provided in relation to these online services, so‐called ‘digital intelligence in overseas Chinese affairs’ (*shuzhi qiaowu*) was applied to transnationally track individual Chinese nationals living overseas.

Consenting to the Chinese state's epidemiological tracking measures through WeChat, Chinese transnationals provided personal data such as daily temperature measurements, thus enabling technology‐enhanced diaspora governance.

Next, we explore how the Chinese diaspora agencies applied WeChat to mediate communication with Chinese in Italy, to discuss the implications of a possible illiberal invasion of privacy and potentially arbitrary diaspora surveillance by the Chinese state.

## WeChat AS A CRUCIAL TRANSNATIONAL TOOL FOR DIASPORA MOBILIZATION

As a ‘super‐sticky’ mega platform (Chen et al., 2018) and a critical infrastructure being indispensable in China's social life with its multiple services (Plantin & de Seta, 2019), WeChat allows for immediate interaction in virtual communities. This messaging app is owned by a private company, Tencent, but is subject to political control by the Chinese state and has over 1.2 billion active monthly users worldwide, of which at least 100 million are outside of China (China Internet Watch Team, 2022; Ryan et al., 2020). By now, WeChat dominates China's mobile social media market, outperforming all the other apps in terms of market share and market value (Chen, 2022).

During the pandemic, transnational Chinese regularly used WeChat for communicating with social networks (Sun & Yu, 2022). As was the case elsewhere in Europe (Zeng, 2020) at the initial outbreak of the virus, many Chinese in Italy organized and transferred donations to Wuhan over the WeChat Pay feature. Later, as the virus spread in Italy, Chinese officials or those acting on behalf of them established a large number of WeChat groups allowing them to liaise directly with individual Chinese in Italy (Haiwai qiaobao, 2020). Online declaration forms for those infected by the virus to be submitted to the Chinese embassy were made available through WeChat groups.

The Chinese embassy and the consulates mobilized ethnic Chinese associations all over Italy to patrol urban areas with dense Chinese populations, and WeChat groups proved effective tools for communication and coordination of these activities. In turn, the zeal of ethnic Chinese associations in enforcing COVID‐19 prevention rules, relying on WeChat groups, in some cases ended up becoming a form of vigilantism. One interviewee (No. 8) recounted that, through WeChat, the president of one Chinese association in the city where she lived tried to control the movements of the Chinese residing in the area, openly soliciting the active support from the participants in the chat. Cases of privacy invasion like this confirm the emergence of unprecedented vigilantism and diasporic control during the pandemic (see also Negro & Hu, 2022).

In areas without strong ethnic associations, Chinese were approached by Chinese representations to set up ad hoc WeChat groups possibly aided by WeChat's ‘People Nearby’ function to reach out to Chinese everywhere in Italy to send them ‘health packages’.

According to our interviewees, registering with specific WeChat groups was mandatory for Chinese professionals and academics working for Chinese or Italian institutions, and for government‐sponsored students. Already existing WeChat groups were reactivated, for example in Prato, where a WeChat group set up in 2019 was reactivated by two Chinese Italians who had run for local election to disseminate information on the situation in China and official documents from the Chinese consulate in Florence. In Rome, information from the Embassy was even conveyed through Chinese supermarkets’ WeChat groups, alongside their special offers. Thus, Chinese supermarket customers contributed to the web of WeChat groups. Interviewees confirmed that they also registered for WeChat groups set up by local Chinese authorities in charge of overseas Chinese affairs in their areas of origin in China that offered medical consultations and psychological assistance.

The case of a young Chinese lawyer helps to understand the extent to which one of the Chinese consulates reached out to local Chinese to organize them in WeChat groups for the purpose of mass mobilization. In April 2020, the Chinese Consulate entrusted the young lawyer as the organizer of a WeChat group for distributing PPE in one region of Italy with few Chinese. As in many other cases, the lawyer perceived this co‐optation as rewarding. To distribute the face masks to local Chinese, the lawyer identified the whereabouts of most of those living in the region and sent the information to the consulate.

The Chinese Consulate [in Florence] gave me the task of creating a WeChat group for distributing masks and disinfectant in Umbria. With my WeChat group, I reached all the families in Umbria, about 350 families. Perhaps a dozen families did not participate in the group, but the information reached them, too, because the news was circulating. I registered all the participants in the WeChat group with their passports, I had to do this to be sure I distributed the masks correctly. And I turned over all the information to the consulate. I distributed all the masks in 4 days. […] The group has never been closed, now 308 families are still in the group, I still keep the WeChat group open because some have questions about the consulate's opening hours, flights to and from China and other information. (Interview No. 3)

In this manner, Chinese official documents and information were shared repeatedly in growing numbers of WeChat groups. Thus, within a short period of time, UFWD, AFROC and Chinese government representatives in Italy were able to involve and mobilize many of the Chinese in Italy. WeChat groups, new and old, based in Italy and in China, directly controlled by Chinese diaspora agencies or set up by ethnic associations associated to the UFWD and private Chinese citizens, and even WeChat groups otherwise used for local commercial activities, were employed or reactivated, apparently without following European data‐protection and privacy laws.

## MOBILIZING AND INTERNALIZING CHINA'S HEGEMONIC DISCOURSE

During the outbreak of COVID‐19, we noticed how Chinese migrants and their descendants residing in Italy actively chose to comply with health regulations and guidelines from China. The case of the Italian Chinese lawyer mentioned above illustrates how China's digitalized pandemic health support and the willingness to accept health monitoring practices became intertwined. At the start of the pandemic, the lawyer had independently started five WeChat groups to address the pandemic, each with some 400 participants, where information collected from both Chinese and non‐Chinese websites and social media was posted. These groups also collected money to buy PPE to send to China. His experience in launching and managing spontaneous WeChat groups with many participants, together with the lack of strong Chinese associations in the region where he lived, was the reason why, later, the lawyer established a new WeChat group in agreement with, and on behalf of, the Chinese Consulate to distribute PPE from China to local Chinese in Italy. In this example, co‐optation of the lawyer by the representatives of the Chinese Party‐state in Italy was intertwined with his active engagement. In other words, Chinese in Italy were not simply the passive recipients of discourses and practices from China but became active participants in developing and expanding digital networks connecting and mobilizing many Chinese in Italy into virtual communities.

Conversely, this case shows the flexible pragmatism of the consulate in identifying and channelling the individual communication skills of an outsider into the Chinese institutions’ cyclopic task of reaching as many Chinese as possible, and entrusting him with responsibilities similar to those of the ethnic Chinese associations linked to the UFWD.

One interviewee recounted how her university in China set up a WeChat group to specifically monitor the small number of teachers abroad, and through this group her health status was checked twice a day. These checks were still being made as late as July 2020. Although the daily health status checks were perceived as invasive, this interviewee and others did not regard the WeChat groups as a form of control; instead, she expressed appreciation for the WeChat groups’ offers of advice and material support. As a result, they regarded the extremely dense WeChat networks that coordinated Chinese nationals in Italy as reflecting primarily voluntary activity, born out of the need for information and help, and not as something imposed by the state.

Interviewees reproduced the Chinese Party‐state's narratives in their articulation of a compassionate and responsible Chinese government. In line with Beijing's ‘mask diplomacy’ to emphasize the image of a benevolent China, one interviewee reiterated the narrative of China's compassion extending worldwide by proudly explaining that China was selling ventilators to Italy at state‐controlled prices, well below the actual market price:

… yes, ventilators were bought [by Italy], they did not come as a donation [from China], but don't forget that they were bought at controlled prices. I looked for information on Chinese sites, there are forums where they wrote how much money must be raised to buy respirators. In one forum there were prices for respirators and the price at the peak of the health crisis was lower than usual. This was thanks to the direct intervention of the Chinese government, which drew up the list of certified companies that could also sell respirators abroad. And the government also decided they had to sell them at a fixed price. They also had to guarantee their quality, or the Chinese government would lose face. We certainly do not want to risk losing face. (Interview No. 5)

Most Chinese in Italy would primarily be exposed to mainstream online news from Chinese media sites in the Chinese language and far less from Italian or international media outlets. One factor contributing to the success of WeChat is the delivery of news stories and information via WeChat official accounts. On WeChat, both Party‐state media and ‘self‐media’, or independently operated accounts, provide mass‐appeal contents far more entertaining than news reports from traditional outlets. One outstanding example of ‘self‐media’ spreading the Party‐state hegemonic discourse is *Guangchazhe*, operated by a non‐official think tank based in Shanghai considered a bastion of pro‐regime opinion leaders (Chen, 2022, p. 94). Highly nationalistic news from the Chinese state media and ‘self‐media’ such as *Guangchazhe* are copied and pasted, according to one interviewee (No. 14), in Chinese‐operated websites in Italy, such as *Weishi* (*Yidali Weishi Chuangmei*) and *Huarenjie*, to reach most of the Chinese in Italy, including the youngest ones. This was confirmed by another interviewee (No. 11) who argued that those in their 20s only read Chinese mainstream news and only trust Chinese websites. In his eyes, these young adults together with first‐generation Chinese migrants, who only read Chinese, are those most likely to support China's nationalistic discourse:

All those who started working when they were young and remained in the Chinese circles are openly nationalists. They are surrounded by people who act as a sounding board for Chinese speech, they always have their mobile phones in their hands and visit only Chinese sites, but Chinese sites invariably give information favourable to China. … Those in their twenties believe that all the answers can be found on Baidu. They are overconfident, the internet makes them believe they are non‐partisan, they are not aware that those who read news only in Chinese are influenced by China, mobile apps shape the way you see the world. (Interview No. 11)

In our ethnographic study, internalizing the Chinese Party‐state's discourse and supporting the Chinese political system were, however, also evident among those fully able to understand the Italian language and with resources to access international media. As discussed above, the Chinese Party‐state invested considerable efforts in shaping a narrative of China being a victim of COVID‐19, but highly competent and a model country in containing the viral outbreak especially compared to liberal democracies in Europe such as Italy. Within this discourse, Chinese state‐dominated media started to mock ‘Western’ countries for being unable to even properly ‘copy homework’ from China, thus positioning China as the teacher when it comes to battling COVID‐19. This catchphrase, which emphasized China's superiority and Western countries’ ineptitude, also went viral among the Chinese in Italy.

An interviewee stated that the Chinese in Italy systematically compared the Italian efforts to combat the pandemic with China's based on national pride in China's achievements:

Chinese people think that China will be the country that leads the world out of the pandemic, there is much pride in China, both in China and among the overseas Chinese. (Interview No. 2)

The process of conforming to the Party‐state was facilitated by increasingly open pride in China's accomplishments. In other words, in the interviewees’ narrative, China's superior handling of the pandemic rested on previously internalized layers of pride in China's economic and technological power.

Interestingly, recurrent patriotic statements were explicitly linked to the pre‐eminence of China's political system. Thus, China's role as a global superpower seems to be a potent element in terms of attracting the support and loyalty of Chinese diasporas. In turn, this sense of superiority boosted a sense of national and cultural homogeneity among Chinese in Italy and was reflected in China‐informed responses to the pandemic in Italy (see below).

Although many Chinese in Italy seemed to internalize the Chinese Party‐state's discourses, a minority of university‐educated young Chinese professionals did not. For instance, during the pandemic, the Wuxu association, founded in Bologna in 2017, translated documents and collected donations to buy PPE to donate to the Wuhan Central Hospital, as other associations did. However, when eliciting donations, they clarified that they would avoid the official channels and instead choose groups of local volunteers to distribute donations; moreover, they made it clear that donations were to be made to ‘the hospital of Dr. Li Wenliang’, the ophthalmologist who raised awareness of early COVID‐19 infection and was accused of scaremongering, and thus distanced themselves from China's official management of the crisis. Eventually, when the Wuxu association was not able to send the money to the hospital in China, at a time when the pandemic was at its peak in Italy, it offered the donations to the Veneto Region, one of the hardest‐hit areas in Italy.

The Wuxu association is different from the typical ethnic Chinese associations in Italy in that it never looked for approval from the Chinese Consulate and established Chinese associations and did not contribute to spread the Chinese narrative as many ethnic Chinese associations in Italy did. One focus of Wuxu was to report in Chinese news outlets on the pandemic situation in Italy and to try to reverse the Chinese widespread narrative that Italy was not good at dealing with the pandemic and needed China's help to ‘sort itself out’ of the pandemic (Interview No. 14). During the first 5 months of 2020, the number of readers accessing articles in the association's WeChat account grew from 600 to 7100 (Interview No. 12).

Focusing on both artistic and social action from below, Wuxu caters almost exclusively to the Chinese students who study or have studied visual arts in Italy. Given its target group, Wuxu is not representative of the majority of university‐educated young Chinese in Italy and remained quite isolated in its positioning vis‐à‐vis China's management of the pandemic.

In summary, with some outstanding exceptions, the COVID‐19 emergency seems to have enabled China's new digitized diaspora governance practices down to the level of the individual Chinese living in Italy by successfully disseminating information, guidelines and news to obtain overseas discursive control. Concurrently, and thanks to the Party‐state's ability to blend compassion with paternalistic control and nationalistic discourses, a large number of the Chinese in Italy chose to actively and proudly comply with being digitally mobilized and monitored as well as having their private data harvested and potentially processed through WeChat.

## COMPARING CHINA'S AND ITALY'S FIGHT AGAINST THE PANDEMIC

The apparent positioning of many Chinese in Italy in line with the Chinese Party‐state was also driven by the national conditions in Italy. In February 2020, Chinese in Italy being acutely aware of the situation in Wuhan were shocked to see that at a moment when they needed PPE and guidance, the Italian government was still unaware of the danger and urgent need for drastic measures. As in most of Europe, in January and early February, episodes of Sinophobia were frequent in Italy with ‘Chinese‐looking’ people verbally and physically attacked (Miyake, 2021). Moreover, the logistical and health‐related problems of those returning from China after the Spring Festival in February were not considered a concern of local Italian governments. For instance, the city administration of Prato, Tuscany, refused to provide quarantine solutions for the approximately 600 workers returning from China to Chinese‐owned factories, and left their care to the ethnic Chinese associations.

Inevitably, Chinese Italians compared China's successful efforts to tame the pandemic and its ability to frame even its worst moments in positive terms, with Italy's inability to quickly address the pandemic, and its much less triumphant narrative. This led many Chinese and their descendants in Italy to consider China the only reliable source of information, guidance and support. As one of our interviewees testified, China has since become the ‘oracle that predicts the future’:

In February, while we were sending face masks to family members in China, half of our Italian friends were not convinced that masks should be worn. They used to say, ‘China is a totalitarian regime, how can you trust what it says?’. They agreed to wear masks only in mid‐March when WHO officially stated that they could limit the infection. This has severely tested the trust of the Chinese in Italian institutions and media; it has prompted almost everyone to just search for info on Chinese media … I myself now rely on Chinese media much more than before. As a result, now all pay much more attention to the Chinese ‘oracle’, an ‘oracle’ that predicts the future. (Interview No. 11)

Against such a background, Chinese in Italy predominantly chose to follow Chinese methods even in highly localized behaviours, such as deciding when to halt activities, lift the local lockdown and send children back to school. Because of their awareness of the seriousness of the situation in Wuhan, and frightened by the episodes of Sinophobia, the Chinese in Italy were the first to close their businesses. By mid‐February, approximately 80% of all Chinese‐owned businesses were closed (Haiwai qiaobao, 2020).

In early May, the Italian government began to lift the 2‐month national lockdown, but a Chinese study conducted at the Chinese PLA General Hospital in Beijing asserted that Italy should not lift the lockdown until August 2020 (QuiFinanza, 2020). Given this, most Chinese shops and restaurants postponed their reopening. Lorenzo Wang, member of the Young Chinese Entrepreneurs in Europe Association, stated, ‘We should wait a little longer … I am not a virologist, but I have closely followed the evolution of COVID‐19 in China and I would recommend greater caution’ (Del Frate & Gasperetti, 2020). He suggested that businesses not resume full activity until August 2020, echoing the Chinese study that proposed the same month for reopening businesses in Italy. In September 2020, Italian institutions decided that children should return to school, but many Chinese living in Italy opposed this, and instead chose to home school.

China also emerged as an authority regarding the COVID‐19 vaccine. While Europe only started authorizing COVID‐19 vaccines at the very end of 2020, voluntary experimental inoculation was available in China months earlier also to Chinese emigrants who could lawfully skip the line because they lived overseas (Ulivelli, 2020). This situation contributed to consolidate China's position as the authority in managing the pandemic among Chinese in Italy.

China's image as a compassionate country with effective pandemic management also became prominent in Italian media. In April 2020, when the pandemic was raging, an Italian cartoon went viral portraying China helping Italy to rise from the ground, while Europe, represented as the death with a scythe, stood aside with its arms crossed (ANI, 2020).

Trust in the CCP and distrust of Italian authorities was strengthened by the differences in the two countries’ political management of the pandemic. As discussed above, China's determination to politicize the pandemic regarding its global role as a superpower in the making was apparent in a well‐orchestrated communication campaign that celebrated the Chinese political system's superiority in fighting the pandemic, and trumpeted China's willingness to help its global allies in dire straits (Hagström & Gustafsson, 2021).

In contrast, in Italy the crisis was politicized only to settle domestic frictions. For example, there was no interest in emphasizing the material help Italy had offered Wuhan in February 2020. Instead, China's assistance to Italy during the pandemic was trumpeted by the Italian media, and emphasized by the Italian foreign minister, who, to strengthen his political position as the first leader in Europe to sign an agreement with China concerning BRI, went so far as to tout ventilators bought from China as aid from China (Buzzi, 2020). Aid from China to fight the pandemic also enjoyed a visibility seven times higher that aid from the United States and Russia in the Italian television and radio programmes (Carrer, 2020).

Meanwhile, by late February, Italian media discourse had started underscoring the rigor and the concerted effort of the Chinese institutions to fight the virus, with a crescendo of appreciation culminating in the depiction of China as a role model. With the arrival of PPE and ventilators from China, the principal Italian economic newspaper declared that Italy was ‘studying the Chinese model’ to fight the pandemic, and that ‘China is transferring its know‐how to us’ (Il sole 24ore, 2020). According to Chinese sources, during a phone call in March 2020 between the Italian prime minister and the Chinese president, the Italian premier stated that China's measures to contain the virus are ‘experiences to learn from’ (Embassy of the People's Republic of China, 2020). Therefore, unlike other European countries, Italy did not distance itself from the Chinese narrative until a new prime minister was appointed in early 2021.

As the pandemic progressed, the representation of the Chinese Italians in Italy changed. They had a rate of COVID‐19 infection well below the national one and primarily through the Chinese associations donated PPE from China to hospitals across Italy and left masks in the mailboxes of blocks of flats in some Italian cities. All this was documented and widely reported in the Chinese media. In May 2020, a population depicted for decades as disembedded (Ceccagno & Salvati, 2020) became a model for dealing with the pandemic, in the words of the mayor of Prato.

A majority of Chinese living in Italy seems to have been exposed to Chinese discourses and Italian ones that to a certain extent overlapped in their identification of China as the global leader, both competent and compassionate. Praised as models when it came to self‐isolation and social responsibility in Chinese and Italian media, they saw themselves as acting in accord with the Chinese Party‐state and righteous participants in its pervasive transnational space invested with essentialized Chinese values and practices.

## CONCLUSION

In this study, we explored how the authoritarian Chinese diaspora state during the COVID‐19 pandemic in 2020 successfully managed to transform a severe health crisis into a unique geo‐political opportunity for transnational nation‐building and power enhancement through diaspora governance based on extensive use of digital technologies. Managing and mobilizing Chinese diasporas by reaching out on the WeChat application platform via Chinese associations and local Chinese diaspora agencies overseas allowed for instant and interactive communication facilitating both liaising and monitoring possibilities.

The COVID‐19 pandemic stranded millions of Chinese migrants and students overseas in epicentres of the pandemic such as Italy and enabled local Chinese diaspora agencies to transform long‐standing overseas institutional structures and overseas connections into all‐encompassing virtual communities. Allowing for immediate communication and interaction in Chinese, the application platform technology of WeChat supported both the distribution of ‘health packages’, health information and individual health monitoring as well as amplifying official Chinese media discourses on transnational unity and solidarity. Simultaneously, WeChat provided a free and easily accessible interactive media platform for the Chinese Party‐state to spread narratives of the CCP as accountable and superior in its management of the virus to consolidate the discourse of the CCP as the legitimate, compassionate and caring ruler of China.

These recent developments of China's digitally supported diaspora governance not only engendered extraterritorial engagement, mobilization and monitoring but also potentially empowered digital surveillance of Chinese residing overseas. Top‐down liaising and monitoring was intertwined with widespread spontaneous, bottom‐up participation and mobilization. In fact, this new digital mode in diaspora governance could not have taken place without the active acceptance and participation of large segments of the Chinese populations overseas. Our case study of Chinese residing in Italy revealed that many exercised their agency in support of the Chinese Party‐state, although critical voices were also uncovered. In general, Chinese in Italy approved of China's digital monitoring practices and actively participated in sharing information and organizing collective action during the pandemic. The WeChat application was in general not regarded as an instrument of surveillance, nor as breaching personal autonomy.

Chinese migrants’ acceptance of intrusive digital technology may be explained mainly by the lack of an Italian alternative to triumphant Chinese discourses reinforcing loyalty to the Chinese Party‐state and nation. In addition, throughout 2020, the Italian government and media in general were, in contrast to other large European countries, outspokenly supportive of China's handling of the COVID‐19 pandemic.

In this study, we have shown how local Chinese diaspora agencies extended their reach by digital means from targeting primarily the Chinese overseas elite to encompass almost all Chinese migrants in an extensive extraterritorial grid system. However, digital diaspora governance was as demonstrated with the case of Chinese diaspora in Italy continuously acted upon and reshaped from below as the pandemic developed into a health and political crisis during 2020.

It awaits to be seen how WeChat will be applied in the future by national and local Chinese diaspora agencies, but transnational data monitoring, management and surveillance of individual Chinese migrants and their descendants in Europe are now readily available to be invoked by Chinese authorities. Our study sheds light on the importance of considering locally embedded digital technologies for diaspora governance, but further research is warranted to explore the deeper implications of the interplay between digital technology and authoritarian diaspora governance in interaction with Chinese populations abroad.

## CONFLICT OF INTEREST

The authors declare no conflict of interest.

## Data Availability

Research data are not shared.

## Footnotes

1 Except for two interviews done by a research assistant. We are grateful to Ding Yujie for her research assistance.

2 The United Front Work Department (UFWD) directly reports to the CCP and is focussed on liaison work with Chinese diasporas among other entities and people outside the CCP. The All‐China Federation of Returned Overseas (AFROC), for example, carries out liaison work with overseas Chinese ethnic associations, Chinese ethnic media and Chinese ethnic schools.
